# Fumonisins at Doses below EU Regulatory Limits Induce Histological Alterations in Piglets

**DOI:** 10.3390/toxins11090548

**Published:** 2019-09-19

**Authors:** Chloé Terciolo, Ana Paula Bracarense, Pollyana C.M.C. Souto, Anne-Marie Cossalter, Léonie Dopavogui, Nicolas Loiseau, Carlos A. F. Oliveira, Philippe Pinton, Isabelle P. Oswald

**Affiliations:** 1 Toxalim (Research Center in Food Toxicology), Université de Toulouse, INRA, ENVT, INP-Purpan, UPS, 31300 Toulouse, France; chloe.terciolo@inra.fr (C.T.); anne-marie.cossalter@inra.fr (A.-M.C.); leonie.dopavogui@inra.fr (L.D.); nicolas.loiseau@inra.fr (N.L.); philippe.pinton@inra.fr (P.P.); 2 Laboratory of Animal Pathology, Universidade Estadual de Londrina, Londrina, PR 86057-970, Brazil; ana.bracarense29@gmail.com; 3 Departamento de Engenharia de Alimentos, Faculdade de Zootecnia e Engenharia de Alimentos, Universidade de São Paulo, Pirassununga, SP 13635-900, Brazil; pollyanasouto@gmail.com (P.C.M.C.S.); carlosaf@usp.br (C.A.F.O.)

**Keywords:** fumonisin B1, toxicity, histopathology, European Union regulatory limits

## Abstract

Fumonisins (FBs) are mycotoxins produced by *Fusarium* species that can contaminate human food and animal feed. Due to the harmful effects of FBs on animals, the European Union (EU) defined a recommendation of a maximum of 5 mg FBs (B1 + B2)/kg for complete feed for swine and 1 µg FBs/kg body weight per day as the tolerable daily intake for humans. The aim of this study was to evaluate the toxicity of dietary exposure to low doses of FBs, including a dose below the EU regulatory limits. Four groups of 24 weaned castrated male piglets were exposed to feed containing 0, 3.7, 8.1, and 12.2 mg/kg of FBs for 28 days; the impact was measured by biochemical analysis and histopathological observations. Dietary exposure to FBs at a low dose (3.7 mg/kg of feed) significantly increased the plasma sphinganine-to-sphingosine ratio. FBs-contaminated diets led to histological modifications in the intestine, heart, lung, lymphoid organs, kidney, and liver. The histological alterations in the heart and the intestine appeared at the lowest dose of FBs-contaminated diet (3.7 mg/kg feed) and in the kidney at the intermediate dose (8.1 mg/kg feed). At the highest dose tested (12.2 mg/kg feed), all the organs displayed histological alterations. This dose also induced biochemical modifications indicative of kidney and liver alterations. In conclusion, our data indicate that FBs-contaminated diets at doses below the EU regulatory limit cause histological lesions in several organs. This study suggests that EU recommendations for the concentration of FBs in animal feed, especially for swine, are not sufficiently protective and that regulatory doses should be modified for better protection of animal health.

## 1. Introduction

Fumonisins (FBs) are a family of mycotoxins produced by *Fusarium* species, especially by *F. verticillioides* and *F. proliferatum*. The different fumonisins identified so far have been grouped in A, B, C and P categories [1]. Among them, fumonisin B1 (FB1), and, to a lesser extent, fumonisin B2 (FB2) are the most abundant and most widely documented. Following consumption of contaminated food or feed, FBs are toxic for humans and animals [2].

Because of its structural similarities to sphingoid bases (sphingosine (So) and sphinganine (Sa)), FB1 interferes with sphingolipids synthesis by inhibiting ceramide synthase [3] increasing the sphinganine to sphingosine ratio (Sa/So ratio) in plasma, tissue, and cells lines [4,5]. Disruption of sphingolipid metabolism inhibits a number of cell processes including cell membrane function, cell growth, cell differentiation, cell injury, and apoptosis [6,7].

In humans, consumption of FBs has been associated with increased incidence of esophageal cancer and the International Agency for Research on Cancer (IARC) classified FB1 and FB2 in group 2B (possible carcinogenic to humans) [8,9]. Regulatory limits have been established in many countries. In the EU, the maximum level for total FBs (FB1 + FB2) ranges from 200 µg/kg of body weight (bw) for processed maize-based foods and baby foods for infants and young children to 2000 µg/kg of bw for unprocessed maize [10]. Recently the European Food Safety Authority (EFSA) established a tolerable daily intake (TDI) for FBs of 1.0 µg/kg bw per day [10].

In animals, high concentrations of FBs cause various clinical signs. FBs elicit nephrotoxicity [2], hepatotoxicity [11], immunotoxicity [12,13], disturbances of the intestinal barrier function [14], and microbiota dysbiosis [15]. Recently, Regnier et al. showed that FB1 hepatotoxicity involved the LXR pathway [16]. This study highlights that FB1 toxicity is associated with negative regulation of LXR. The nuclear factor LXR is known to play a role in lipid metabolism and to participate to cholesterol transport [16]. More specific adverse effects have also been reported including leukoencephalomalacia in horses, pulmonary edema and cardiac dysfunction in swine [17]. These harmful effects led the EU to establish guidance values for FBs in products intended for use as animal feed. The guidance values for FBs are fixed at 60 mg/kg for maize and maize products and at 5 mg/kg for complete swine feed [18,19,20]. Recently EFSA revised this value and identified a NOAEL of 1 mg FB1/kg for swine feed [10].

The aim of this study was to investigate the effects of diets contaminated by low concentrations of FBs, including a concentration below the current EU regulation, on piglets. Because of their cereal diet, swine may be exposed to high concentrations of FBs, and they are particularly sensitive to FB1 [21]. In addition, thanks to their anatomical, genetic, and pathophysiological similarities [22,23], they are a species of choice as a biomedical model for human toxicology. We thus investigated histological changes caused by FBs in different tissues (heart, intestine, kidney, liver, mesenteric lymph nodes, spleen, and lung) in swine. We focused on organs already known to be altered by exposure to FBs. Indeed, lung and heart are involved in pulmonary edema [17]; kidney and liver are two major targets of FBs; the intestine acts as a barrier to food contaminants and lymphoid organs making it possible to investigate the immune response induced by exposure to FBs.

## 2. Results

### 2.1. Effects of FBs on Animal and Organ Weights

Piglets fed diets contaminated with increasing doses of FBs were weighed once a week for four weeks. As shown in Table 1, piglet body weight increased continuously from week 1 (2.6–2.9 kg) to week 4 (6.6–7.1 kg), and no difference in body weight was observed between animals receiving the different diets. At the end of the experiment, spleen, liver, lung, kidney, and heart were weighed and no significant difference was observed between the organ weights of the animals (Table 2).

### 2.2. Effects of FBs on Immune Response and Biochemical Parameters

The concentration of the different immunoglobulin subsets (IgG and IgA) was assessed after immunization against ovalbumin, and increased production of immunoglobulin subsets was observed over the course of the experiment. By contrast, ingestion of diets contaminated with increasing doses of FBs altered neither the total plasma concentration (data not shown) nor the specific immune response to the vaccinal antigen (data not shown). 

**Figure 1:**
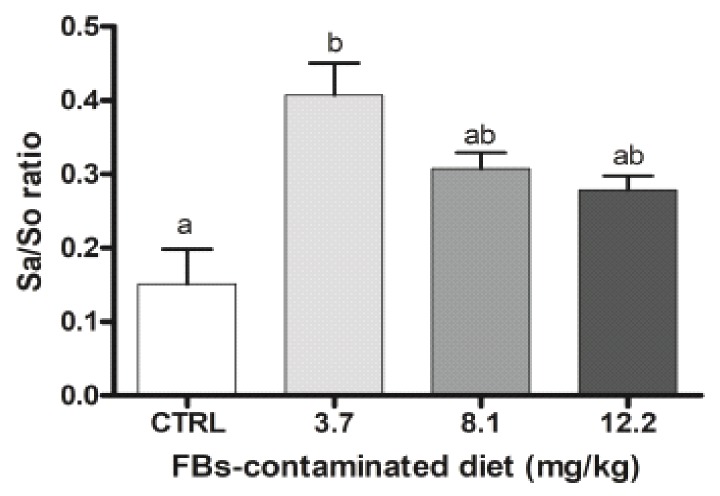
Mean serum sphinganine-to-sphingosine ratio (Sa/So) of piglets receiving fumonisins (FBs)-contaminated feed (0, 3.7, 8.1, or 12.2 mg/kg). Samples were collected from individual piglets (*n* = 3 to 6) after 28 days of exposure to different diets. Error bars represent the standard error of the mean. ^a,b,c^ mean values with different letters are statistically different (*p* < 0.01).

Serum levels of sphinganine (Sa) and sphingosine (So) in each FBs-contaminated diet were measured at the end of the experiment, and their ratio was calculated (Figure 1). The Sa/So ratio is a sensitive biomarker of fumonisin exposure in swine. As expected, ingestion of FBs induced a slight accumulation of Sa and to a lesser extent of So. The Sa/So ratio of animals treated with 3.7 mg of FBs/kg of feed was 1.6-fold higher in plasma than in control animals (*p* < 0.01). By contrast, higher doses of FBs (8.1 and 12.2 mg of FBs/kg of feed) did not significantly increase the Sa/So ratio (*p* > 0.01).

Blood samples were taken once a week throughout the experiment to investigate the effects of FBs-contaminated diet on plasma biochemical parameters. Only ingestion of the diet contaminated with 12.2 mg/kg of FBs led to changes in plasma during the experiment. An increase in the concentration of triglycerides was observed at d14 (*p* < 0.05). The plasma concentration of urea was higher at d28 (*p* < 0.05), suggesting alterations in the liver and kidney. There was also a slight increase in the creatinine plasma level over the course of the experiment (*p* > 0.05). These results show that exposure to 12.2 mg/kg of FBs-contaminated diet induced hepato- and nephrotoxicity in the piglets tested.

### 2.3. Effects of FBs on Histological Parameters

Histological changes were found in all the tissues evaluated in piglets fed FBs-contaminated diets. The lesions observed in the tissues (heart, intestine, kidney, liver, lymphoid organs, and lung) were mild to moderate in piglets fed FBs-contaminated diets (3.7, 8.1, 12.2 mg/kg feed). The ingestion of 12.2 mg FBs/kg led to a significant increase in the lesional scores in all tissues, compared to piglets fed the control diet.

#### 2.3.1. Heart

**Figure 2:**
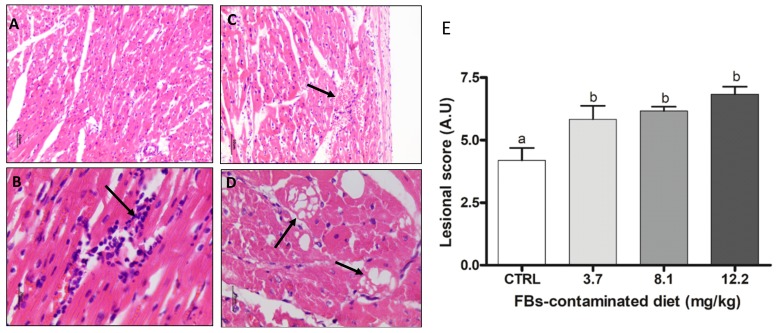
Histological aspects of the heart of pigs submitted to different treatments. (**A**) Normal heart. Control. HE. Bar 40 µm. (**B**) Lymphocytic inflammatory infiltrate (arrow). FBs 3.7 mg/kg. HE. Bar 20 µm. (**C**) Focal hemorrhage in myocardium (arrow). FBs 8.1 mg/kg.HE. Bar 50 µm. (**D**) Multifocal myocyte cytoplasmic vacuolation (arrows). FBs 12.2 mg/kg. HE. Bar 20 µm. (**E**) Lesional score after histological examination according to the occurrence and the severity of lesions. Values are means with standard errors of the mean represented by vertical bars (*n* = 6 animals). ^a, b^ mean values with different letters are statistically different (*p* < 0.05).

In the heart, 3.7 mg/kg FBs-contaminated diet increased the lesional score. The main histological observations associated with the lesional score were hemorrhage and lymphocyte inflammatory infiltrate (Figure 2).

#### 2.3.2. Intestine

**Figure 3:**
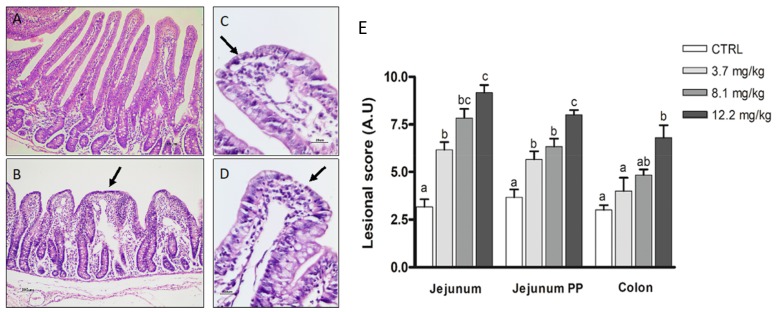
Histological aspects of the jejunum of pigs submitted to different treatments. (**A**) Normal villi in jejunum. Control. HE. Bar 100 µm. (**B**) Villi atrophy and fusion (arrow). FBs 3.7 mg/kg. HE. Bar 100 µm. (**C**) Enterocyte flattening (arrow). FBs 8.1 mg/kg.HE. Bar 20 µm. (**D**) Enterocyte apical necrosis (arrow). FBs 12.2 mg/kg. HE. Bar 20 µm. (**E**) Lesional score after histological examination according to the occurrence and the severity of lesions. Values are means with standard errors of the mean represented by vertical bars (*n* = 6 animals). ^a,b,c^ mean values with different letters are statistically different (*p* < 0.05).

Three segments of the intestine were analyzed (jejunum, jejunum with Peyer’s patches and colon). In jejunum, flattening of enterocytes and apical necrosis of villi were the most frequent histological changes. These changes were associated with a dose-dependent increase in the lesional score. These histological changes were also observed in the colon but were only significant in piglets fed the highest dose of FBs (12.2 mg/kg) (Figure 3).

#### 2.3.3. Kidney

**Figure 4:**
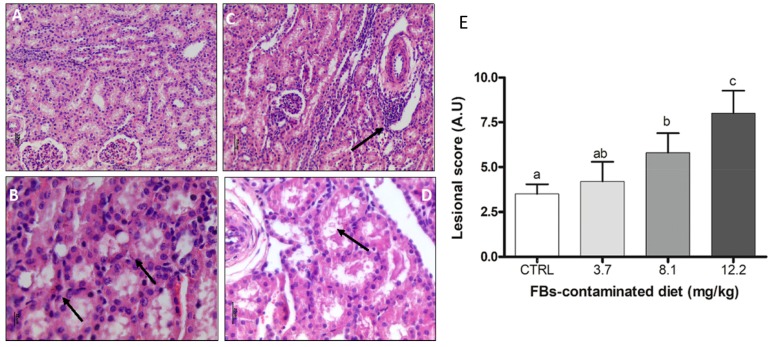
Histological aspects of the kidney of pigs submitted to different treatments. (**A**) Normal kidney. Control. HE. Bar 30 µm. (**B**) Nuclear tubular epithelial cell vacuolation (arrow). FBs 3.7 mg/kg. HE. Bar 10 µm. (**C**) Lymphocytic interstitial infiltrate (arrow). FBs 8.1 mg/kg.HE. Bar 50 µm. (**D**) Focal tubular necrosis (arrow) and luminal cell debris. FBs 12.2 mg/kg. HE. Bar 20 µm. (**E**) Lesional score after histological examination according to the occurrence and the severity of lesions. Values are means with standard errors of the mean represented by vertical bars (*n* = 6 animals). ^a,b,c^ mean values with different letters are statistically different (*p* < 0.05).

Plasma parameters revealed nephrotoxicity in swine feed contaminated with 12.2 mg/kg FBs. To evaluate the effects of FBs on tissue, we analyzed histological parameters in the kidney. A dose-dependent increase in the lesional score was observed. Lesions in the kidney were mild associated with necrosis. Degenerative changes in tubular epithelial cells (vacuolation of cytoplasm and nucleus) with interstitial infiltration of lymphocytes were observed in piglets exposed to 8.1 mg/kg of feed (Figure 4). Nephrotoxicity was observed in the kidney of piglets fed the diet contaminated with 12.2 mg FBs, while significant histological alterations in this tissue appeared in piglets fed the diet contaminated with 8.1 mg/kg FBs.

#### 2.3.4. Liver

**Figure 5:**
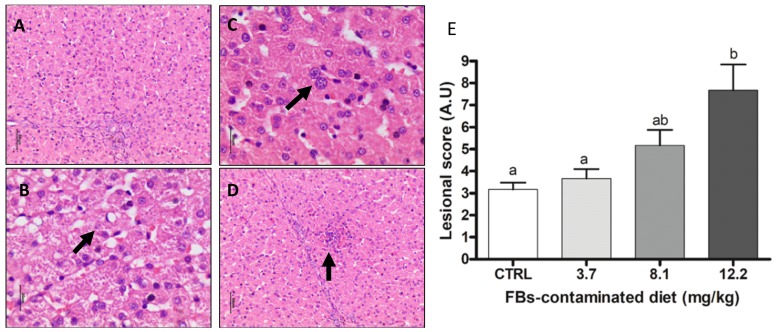
Histological aspects of liver of pigs submitted to different treatments. (**A**) Normal liver. Control. HE. Bar 50 µm. (**B**) Nuclear hepatocyte vacuolation (arrow). FBs 3.7 mg/kg. HE. Bar 20 µm. (**C**) Hepatocyte megalocytosis (arrow). FBs 8.1 mg/kg.HE. Bar 20 µm. (**D**) Focal hepatocyte necrosis (arrow). FBs 12.2 mg/kg. HE. Bar 50 µm. (**E**) Lesional score after histological examination based on the occurrence and the severity of lesions. Values are means with standard errors of the mean represented by vertical bars (*n* = 6 animals). ^a,b^ mean values with different letters are statistically different (*p* ≤ 0.05).

Biochemical analysis revealed alterations in liver function in piglets fed the diet contaminated with 12.2 mg/kg of FBs. The main histological lesions observed in the liver were megalocytosis, nuclear and cytoplasmic vacuolization of hepatocytes. Only piglets that received 12.2 mg FBs/kg feed showed a significant increase in the lesional score (Figure 5). Together, biochemical parameters and histological analyses indicated hepatotoxicity at a dose of 12.2 mg/kg FBs-contaminated diet.

#### 2.3.5. Lymphoid Organs

**Figure 6:**
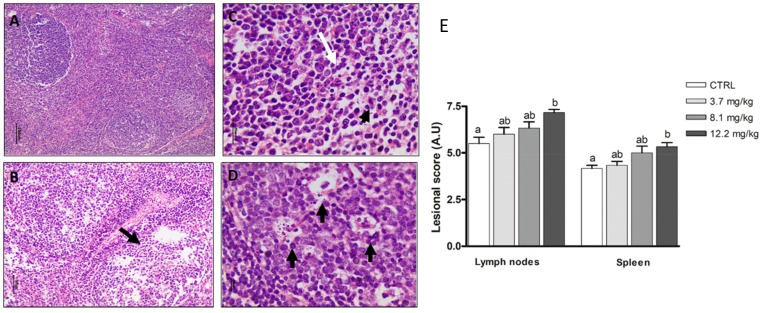
Histological aspects of lymphoid organs of pigs submitted to different treatments. (**A**) Normal lymph node. Control. HE. Bar 100 µm. (**B**) Lymphoid depletion (arrow). FBs 3.7 mg/kg. HE. Bar 50 µm. (**C**) Mild apoptosis in lymphocytes (arrow) and mitosis figures (arrowhead). FBs 8.1 mg/kg.HE. Bar 20 µm. (**D**) Multiple apoptotic bodies in lymphoid follicles (arrows). FBs 12.2 mg/kg. HE. Bar 10 µm. (**E**) Lesional score after histological examination based on the occurrence and the severity of lesions. Values are means with standard errors of the mean represented by vertical bars (*n* = 6 animals). ^a, b^ mean values with different letters are statistically different (*p* ≤ 0.05).

Like the liver, the spleen and mesenteric lymph nodes showed a significant increase in the lesional score in piglets fed the highest dose of FBs (*p* < 0.05). Changes were mild and were characterized by the presence of lymphocytic apoptotic bodies and follicular depletion (Figure 6).

#### 2.3.6. Lung

**Figure 7:**
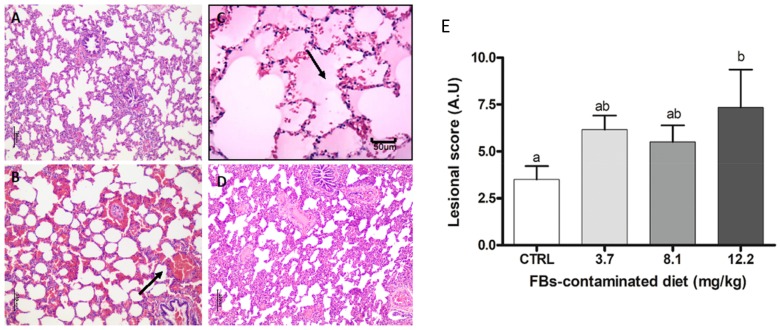
Histological aspects of the lung of pigs submitted to different treatments. (**A**) Normal lung. Control. HE. Bar 100 µm. (**B**) Alveolar hemorrhage (arrow). FBs 3.7 mg/kg. HE. Bar 100 µm. (**C**) Alveolar edema and congestion (arrow). FBs 8.1 mg/kg.HE. Bar 100 µm. (**D**) Diffuse interstitial pneumonia. FBs 12.2 mg/kg. HE. Bar 50 µm. (**E**) Lesional score after histological examination based on the occurrence and severity of the lesions. Values are means with standard errors of the mean represented by vertical bars (*n* = 6 animals). a,b,c mean values with different letters are statistically different (*p* ≤ 0.05).

The changes to the lung consisted of alveolar edema and hemorrhage. As demonstrated by the lesional scores, lung lesions were only observed in piglets fed the 12.2 mg/kg FBs-contaminated diet (Figure 7).

These results show that exposure to FBs led to specific changes that varied with the organ. Some organs were shown to be more sensitive than others to the presence of FBs in the diet (kidney, heart, and jejunum). More surprisingly, changes in the heart and the jejunum appeared at doses below the EU regulatory limit.

## 3. Discussion

The FBs-contaminated diets used in this study did not cause feed refusal and did not interfere with animal growth. These results are consistent with previously published data [21,24,25,26]. Indeed, in most experiments on swine, performances (growth and mortality rate) were only impaired after ingestion of feed contaminated by doses higher than 100 mg FB1/kg feed for four to eight weeks, lower doses slightly modified or did not modify performances [10]. 

By contrast, we observed that dietary exposure at 3.7 mg FBs/kg of feed caused a significant increase in the sphinganine to sphingosine (Sa/So) ratio. FBs disrupt sphingolipid metabolism by inhibiting ceramide synthase leading to an increased Sa/So ratio in tissues and body fluids [5,27].

As known from several publications, inhibition of ceramides decreases hepatic triglycerides and hepatic lipogenesis [28,29] and destabilizes fatty acid transport protein able to absorb plasmatic lipids from lipoproteins or produce and stabilize lipid vesicules. Moreover, higher concentration in FB1 is also known to inhibit gene expression of de novo lipogenesis [30]. Our results on the plasmatic ratio of Sa/So, which are higher for the lowest concentration of FBs/kg than for the highest, could be explained by the fact that higher concentration of FBs disturb much more lipid composition (ceramides, triglycerides, cholesterol, cholesterol ester and sphingoïd bases) of plasmatic lipoproteins. Indeed, sphingoïd bases being part of the lipid vesicle stability [31], we can assume lipoproteins include much more sphinganine at low concentration of FBs when lipogenesis de novo is not disturbed than at higher concentration. At least, as shown in Table 3, the transient and small increase of plasmatic triglycerides at high concentration of FBs observed after only 14 days of FBs exposure could be explained by the induction of the lipolysis, as whole-body response, during the early stage of hepatic lipogenesis inhibition. 

The change in the serum Sa/So ratio is a sensitive marker for the assessment of adverse effects of FBs [32]. In line with our data, an increase in Sa/So ratio was also observed in swine exposed to 2 mg FBs/kg of feed [4,32,33]. The increase in this ratio evaluates the exposure of animals to FBs and is particularly useful in the study of recommended values of exposure/toxicity to FBs. In the present study the increased Sa/So ratio was associated with histological alterations in several organs.

Fumonisin toxicosis in swine is characterized by lung, liver, and heart injuries. Lung lesions such as hemorrhage and alveolar edema were observed in the present study. There was a two-fold increase in the lesional score in the lungs of piglets fed the diet contaminated with 12.2 mg FBs/kg feed. Histological alterations in the lung may be associated with pulmonary porcine edema [2]. This pathogenesis is partially explained by accumulation of sphingosine in the heart that blocks L-type calcium channels thereby reducing calcium release and cardiac contractility. Furthermore, left side cardiac insufficiency and the drop-in mean arterial pressure cause a pulmonary edema [2]. In the present study, the heart lesional score increased 1.5- and 1.75-fold in piglets fed the diet contaminated with 3.7 and 12.2 mg FBs/kg feed, respectively. The main histological alterations observed in the heart were the presence of hemorrhage and lymphocyte inflammatory infiltrate. Clinical changes such as hypertrophy of the heart and of the pulmonary arteries have been observed in swine fed very high doses of FBs (100–193 mg FB1/kg feed for 93 days) [34,35]. Our result indicates that exposure to a very low dose of FBs (3.7 mg/kg feed) also induces histological alterations in the heart but that this low dose is not high enough to cause pulmonary edema.

Dietary exposure to FB1 results in dose- and time-dependent injuries to the liver and kidney that can also be detected by elevated serum biomarkers [17]. The bioavailability of FB1 through oral administration is very low (less than 10%), and its accumulation in tissues is highest in the liver and the kidney [27,36]. In the present study, the enzymatic liver markers (AST, ALT, and ALP) were not influenced by ingestion of FBs at a dose of 3 to 12 mg/kg feed. This is consistent with literature reports of liver injuries upon exposure to high concentrations of toxins (100–175 mg FBs/kg feed) [32]. By contrast, an increased level of plasma urea, a marker of renal injury, was observed in piglets exposed to 12.2 mg FBs/kg feed; while renal histological alterations (1.7-fold increase in the lesional score) were already observed upon exposure to 8.1 mg/kg of FBs. The increase in plasma urea was correlated with an increase in the production of sphingosine-1-phosphate, which may be up-regulated after exposure to FBs [37]. Little information is available in the literature concerning the renal biomarkers (creatinine, urea). However, in a study on 5-week old piglets that had received 6 mg/kg FBs, only the creatinine plasma level was higher than in the control [38]. Increases in serum creatinine were also reported in a study 4-week old piglets fed with 8 mg FBs/kg of feed [39].

After ingestion of contaminated feed, the intestine is exposed to high doses of FBs, and our group and others have shown that ingestion of food contaminated with FBs causes various intestinal disorders [7,40]. The present study revealed necrosis and flattening of apical enterocytes in the small intestine after exposure to a very low dose (3.7 mg FBs /kg feed). Due to its distal localization, the colon is less exposed and less sensitive than the small intestine. Lesions in the colon were only observed in piglets exposed to the highest dose (12.2 mg FBs/kg feed). Histological alterations in the small intestine have already been reported at different concentrations of FBs. Bracarense et al. reported mild to moderate intestinal lesions associated with a 4-fold increase in the lesional score in animals fed with 5.9 mg FBs/kg [7]. The same results were obtained in a study by Grenier et al. in animals that received 2 mg of FBs/kg bw/day by gavage [40]. Piva et al. reported histological alterations in weaning piglets exposed to a diet containing 30 mg of FB1/kg of feed [41]. Another study observed necrosis in 50% of cells in the small intestine of mice fed a diet contaminated with 10 ppm of FB1 for 60 days [42]. 

## 4. Conclusions

From the point of view of swine health, our data show that feed contaminated at a dose of 3.7 mg/kg feed, i.e., below the regulatory limit of 5 mg FB1/kg feed, has deleterious effects on the heart and intestine of piglets. This suggests that the current regulatory limit is not sufficiently protective. Recently however, a NOAEL of 1 mg FB1/kg feed that did not cause clinical signs or significant performance impairment after a short period (8 weeks) [18] or long period (20 weeks) of exposure [19] was identified by EFSA. It would thus be of interest to repeat our experiment with the lower dose to determine the NOAEL for deleterious effects in the heart and intestine of swine in order to verify if the new health-based guidance value proposed by EFSA in fact ensures the safety of pig feed.

From the point of view of human health, this study confirms the sensitivity of pigs to FBs. Considering the weight of the animals, the present study established a LOAEL of 3.7 mg/kg corresponding to 148 µg/kg bw per day. This LOAEL is in the range of the BMDL (100 µg/kg bw per day) obtained in mice, which is used by EFSA to set the tolerable daily intake for human [43].

## 5. Materials and Methods

### 5.1. Animals

The experimental protocol was performed as previously described [44]. The experiment using swine was conducted with the approval of the Ethics committee of Pharmacology-Toxicology of Toulouse-Midi-Pyrénées in animal experimentation (Toxcométhique), TOXCOM/0018/IO PP (Date of approval: 18 January 2013), in accordance with the European Directive on the protection of animals used for scientific purposes (Directive 2010/63/EU). The study animals were twenty-four 28-day-old weaned castrated male piglets allocated in four groups. Feed and water were provided ad libitum throughout the experiment.

### 5.2. Experimental Diets

FBs used in the experiment were produced by the Veterinary Medical Diagnostic Laboratory, University of Missouri–Columbia as previously described [40,41]. The culture material analyzed for FB1 and FB2 revealed concentrations of 1800 mg FB1/g and 450 mg FB2/g. Fumonisin culture material was added to the basal diet in appropriate amounts to obtain the required levels of FB1. Diets were prepared at INRA facilities in Rennes (France) and formulated according to energy amino acid requirements for piglets as already described [29,42]. The detailed composition is listed in Table 4. After preparation, the concentrations of mycotoxins in the experimental diets were analyzed by LABOCEA (Ploufragan, France), as previously described [45]. Briefly, experimental diets were ground and sifted through a 0.5 mm particle size filter. Five g of sieved samples were extracted by reversal agitation with acetonitrile/water. After centrifugation, the aqueous phase was evaporated and the dry residue was dissolved in 0.01% acetic acid methanol (2/1, *v*/*v*) and submitted to HPLC using Hewlett Packard type 1100 (Hewlett Packard, Eybens 38, France). The detection was performed with a quadrupole tandem mass spectrometer API 4000 (Applied Biosystems, Foster City, CA, USA). Deoxynivalenol and ochratoxin A were found to be naturally present in the cereals used, resulting in concentrations of 0.025, 0.006 m/kg feed, respectively. All other mycotoxins, including aflatoxins, T-2 toxin, HT-2 toxin, and the ergot alkaloids were below the limit of detection (0.004 mg/kg, 0.01 mg/kg, 0.01 mg/kg and 0.01 mg/kg, respectively). The concentrations of FB1 in final diets were 0, 3.1, 6.1, and 9.0 mg/kg; and the one of FB2 0, 0.6, 2.0, and 3.2 mg/kg respectively.

### 5.3. Experimental Design and Sample Collection

Animals received experimental diets for a period of 28 days. Animals were observed daily and weighed weekly. Once a week, blood samples were taken from the external jugular vein of all the animals for antibody and biochemical analyses. Plasma biochemistry was performed at GenoToul-Anexplo platform (Toulouse, France) using a Pentra 400 Clinical Chemistry benchtop analyzer (Horiba, Les Ulis, France). The total concentration of the immunoglobulin subsets was measured by ELISA as previously described [46].

The ratio of the sphingoid bases sphinganine to sphingosine (Sa/So) was measured in the plasma after a regular Bligh and Dyer extraction and analysis as previously described [47].

At the end of the experiment, piglets were subjected to electrical stunning and euthanized by exsanguination. Samples of jejunum, jejunum with Peyer’s patches, colon, lung, liver, kidney, heart, spleen, and mesenteric lymph nodes were fixed in 10% buffered formalin (Sigma, Saint-Quentin Fallavier, France) for histopathological evaluation.

### 5.4. Histopathological Assessment

Tissue samples were dehydrated using graded alcohols (70, 80, 90, and 96%), diaphanized in xilol, and embedded in paraffin wax. Sections of 5 µm were stained with hematoxylin-eosin (HE) for histopathological evaluation. The histological changes were evaluated, and a lesional score was established for each organ (Table 5) based on previous studies [7,34,44,48]. The lesional score was established based on the degree of severity (severity factor) and the extent of each criterion (according to intensity or observed frequency, scored from 0—absent, 1—mild, 2—moderate, and 3—severe; Mild, moderate, and severe are criteria for the intensity of lesions (degree of intensity) using clinical practice guidelines) (Table 5). For each criterion, the score of the extent was multiplied by the severity factor. Villi height was measured randomly on 30 villi using an image analysis software (Motic Image Plus, Motic Instruments, Richmond, BC, Canada). 

### 5.5. Statistics

Histological and biochemical data were measured on six animals per group and statistically analyzed by ANOVA followed by Tukey’s test. The operations were performed in GraphPad Prism statistical software version 4 (GraphPad Software, San Diego, CA, USA).

## Figures and Tables

**Table 1:** Body weight gain in piglets fed 0, 3.7, 8.1, or 12.2 mg fumonisins (FBs)/kg feed (*n* = 6/group).

Body Weight Gain (kg)	Diet (FBs Contamination, mg/kg Feed)			
	Control	3.7	8.1	12.2
Week 1	2.9 ± 0.2 ^a^	2.9 ± 0.3 ^a^	2.8 ± 0.4 ^a^	2.6 ± 0.2 ^a^
Week 2	3.7 ± 0.7 ^a^	3.9 ± 0.6 ^a^	5.6 ± 0.4 ^a^	4.1 ± 0.4 ^a^
Week 3	4.8 ± 0.9 ^a^	6.4 ± 0.5 ^a^	5.7 ± 0.3 ^a^	6.0 ± 0.6 ^a^
Week 4	6.7 ± 0.3 ^a^	6.6 ± 0.9 ^a^	6.7 ± 0.3 ^a^	7.1 ± 0.3 ^a^

Results are expressed as the mean ± SE of 6 animals. ^a^ mean values with the same letter are not statistically different (*p* > 0.05).

**Table 2:** Organ weights of piglets fed 0, 3.7, 8.1 or 12.2 mg FBs/kg feed (*n* = 6/group).

Organ Weight (g)	Diet (FBs Contamination, mg/kg Feed)			
	Control	3.7	8.1	12.2
Spleen	74.7 ± 5.9 ^a^	75.9 ± 4.2 ^a^	72.0 ± 4.3 ^a^	62.0 ± 3.7 ^a^
Liver	962 ± 81 ^a^	906 ± 54 ^a^	1030 ± 23 ^a^	948 ± 40 ^a^
Lung	373 ± 28 ^a^	381 ± 12 ^a^	352 ± 22 ^a^	382 ± 32 ^a^
Kidney	94.9 ± 3.7 ^a^	90.4 ± 5.7 ^a^	93.6 ± 11.6 ^a^	101.7 ± 6.1 ^a^
Heart	211 ± 12 ^a^	170 ± 33 ^a^	204 ± 9 ^a^	178 ± 9 ^a^

Results are expressed as the mean ± SE of 6 animals. ^a^ mean values with the same letters are not statistically different (*p* > 0.05).

**Table 3:** Serum biochemical analysis of piglets fed 0, 3.7, 8.1, or 12.2 mg FBs/kg feed (*n* = 6/group).

Biochemical Parameters		Diet (FBs Contamination, mg/kg Feed)			
		Control	3.7	8.1	12.2
Triglycerides (mmol/L)	day 14	0.4 ± 0.1 ^a^	0.4 ± 0.2 ^a^	0.4 ± 0.1 ^a^	0.6 ± 0.2 ^b^
	day 28	0.5 ± 0.1 ^a^	0.3 ± 0.2 ^a^	0.4 ± 0.1 ^a^	0.5 ± 0.1 ^a^
Urea (mmol/L)	day 14	3.2 ± 1.2 ^a^	3.03 ± 1.1 ^a^	2.4 ± 0.7 ^a^	2.9 ± 0.9 ^a^
	day 28	2.6 ± 0.6 ^a^	2.4 ± 0.6 ^a^	2.5 ± 0.3 ^a^	4 ± 0.9 ^b^
Creatinine (µmol/L)	day 14	79.4 ± 7 ^a^	78.1 ± 16.3 ^a^	82.22 ± 6.3 ^a^	86.2 ± 5.5 ^a^
	day 28	82.7 ± 20 ^a^	71.7 ± 28.9 ^a^	84.4 ± 30.3 ^a^	100.5 ± 35.1 ^a^

^a,b^ mean values with different letters are statistically different (*p* < 0.05).

**Table 4:** Composition of the experimental diet.

Ingredient (%)	
Wheat	47.50
Soybean meal	24.30
Barley	22.90
Calcium phosphate	1.12
Calcium carbonate	1.00
Vitamin and mineral premix ^a^	0.50
Vegetable oil	1.40
Sodium chloride	0.40
Phytase	0.01
Lysine	0.465
Methionine	0.165
Threonine	0.195
Tryptophan	0.045
Composition ^b^	
Starch (g)	476.8
Crude protein (g)	218.3
Crude fiber (g)	37.5
Ca (g)	10.5
P (g)	6.5
K (g)	8.7
Net energy (MJ)	15.6

^a^ Vitamin A, 2,000,000 IU/kg; vitamin D3, 400,000 IU/kg; vitamin E, 4000 mg/kg; vitamin C, 8000 mg/kg; vitamin B1, 400 mg/kg; vitamin K3, 400 mg/kg; iron, 20,000 mg/kg; copper, 4000 mg/kg; zinc, 20,000 mg/kg; manganese, 8000 mg/kg. ^b^ corresponding to 1000 g dry matter/kg.

**Table 5:** Establishment of the lesional score for histological alterations.

	Criteria (Severity Factor)	Maximum Total Score
Heart	Edema (2)	39
	Hemorrhage (2)	
	Congestion (1)	
	Inflammatory infiltrate (2)	
	Myocyte hypertrophy (1)	
	Myocyte degeneration (2)	
	Necrosis (3)	
Intestine	Villi atrophy (2)	39
	Villi fusion (2)	
	Lymphatic dilation (1)	
	Edema (2)	
	Enterocyte flattening (2)	
	Cytoplasmic vacuolation (1)	
	Necrosis (3)	
Kidney	Congestion (1)	27
	Inflammatory infiltrate (2)	
	Cell debris (1)	
	Cytoplasmic vacuolation (1)	
	Nuclear vacuolation (1)	
	Necrosis (3)	
Lymph nodes and spleen	Lymphoid hyperplasia (1)	36
	Lymphoid depletion (1)	
	Histiocytosis (2)	
	Inflammatory infiltrate (2)	
	Apoptosis (1)	
	Mitosis (1)	
	Necrosis (3)	
Liver	Trabecular disorganization (1)	33
	Inflammatory infiltrate (1)	
	Cytoplasmic vacuolation (1)	
	Nuclear vacuolation (1)	
	Megalocytosis (2)	
	Apoptosis (2)	
	Necrosis (3)	
Lung	Edema (2)	21
	Inflammatory infiltrate (2)	
	Hemorrhage (2)	
	Congestion (1)	

Maximum total score was obtained by the multiplication of the maximum intensity level of the lesions by the maximum severity factor for each organ.
